# Disease progression in patients with the restrictive and mixed phenotype of Chronic Lung Allograft dysfunction—A retrospective analysis in five European centers to assess the feasibility of a therapeutic trial

**DOI:** 10.1371/journal.pone.0260881

**Published:** 2021-12-23

**Authors:** Jens Gottlieb, Geert M. Verleden, Michael Perchl, Christina Valtin, Alexander Vallee, Olivier Brugière, Carlos Bravo

**Affiliations:** 1 Dept. of Respir. Medicine OE 6870, Hannover Medical School, Hannover, Germany; 2 Biomedical Research in Endstage and Obstructive Lung Disease Hannover (BREATH), Member of the German Center for Lung Research (DZL), Hannover, Germany; 3 Dept Respir. Med, Lung Transplant Unit, University Hospital Gasthuisberg, Leuven, Belgium; 4 Department of Cardiology, Section for Lung transplantation, Rigshospitalet, Copenhagen University Hospital, Copenhagen, Denmark; 5 Délégation à la Recherche Clinique et à l’Innovation, Hôpital Foch, Suresnes, France; 6 Service de Transplantation Pulmonaire et Centre de compétence de la Mucoviscidose, Hôpital Foch, Suresnes, France; 7 Servei de Pneumologia, Hospital Universitari Val d’Hebron, Barcelona, Spain; Medical University of Gdansk, POLAND

## Abstract

**Background:**

Chronic Lung Allograft Dysfunction (CLAD) is a major obstacle for long term survival after lung transplantation (LTx). Besides Bronchiolitis Obliterans Syndrome, two other phenotypes of CLAD, restrictive allograft syndrome (RAS) and mixed phenotype, have been described. Trials to test in these conditions are desperately needed and analyzing natural outcome to plan such trials is essential.

**Methods:**

We performed a retrospective analysis of functional outcome in bilateral LTx recipients with RAS and mixed phenotype, transplanted between 2009 and 2018 in five large European centers with follow- up spirometry up to 12 months after diagnosis. Based on these data, sample size and power calculations for randomized therapeutic trial was estimated using two imputation methods for missing values.

**Results:**

Seventy patients were included (39 RAS and 31 mixed phenotype), median 3.1 years after LTx when CLAD was diagnosed. Eight, 13 and 25 patients died within 6, 9 and 12 months after diagnosis and a two patients underwent re-transplantation within 12 months leading to a graft survival of 89, 79 and 61% six, nine and 12 months after diagnosis, respectively. Observed FEV_1_ decline was 451 ml at 6 months and stabilized at 9 and 12 months, while FVC showed continuous decline. Using two methods of imputation, a progressive further decline after 6 months for FEV1 was noted.

**Conclusion:**

The poor outcome of these two specific CLAD phenotypes suggests the urgent need for future therapeutic randomized trials. The number of missing values in a potential trial seems to be high and most frequently attributed to death. Survival may be used as an endpoint in clinical trials in these distinct phenotypes and imputation techniques are relevant if graft function is used as a surrogate of disease progression in future trials.

## Introduction

Chronic Lung Allograft Dysfunction (CLAD) continues to be a major obstacle for long term survival after lung transplantation (LTx) [1]. The incidence of CLAD varies between 34% and 49% 5 years after transplantation [2, 3]. Patients affected by CLAD have a poor graft survival with the majority dying from respiratory failure [4]. Unfortunately there are still no convincing treatment options available if CLAD occurs. There is an urgent need to test new or known drugs in randomized controlled trials in this specific population.

CLAD is an umbrella term of different phenotypes, characterized by spirometry, imaging and measurement of lung volumes as described in the most recent ISHLT consensus paper [1]. Restrictive allograft syndrome (RAS), along with the mixed phenotype were recently defined as distinct phenotypes of CLAD [5] in contrast to bronchiolitis obliterans syndrome (BOS) [1]. RAS as well as the mixed phenotype are characterized by persistent opacities on chest computed tomography and loss of lung volume as defined by total lung capacity. The mixed phenotype has an additional obstructive pattern on spirometry while the RAS phenotype is purely restrictive. Both entities together compromise 14 to 26% of all CLAD-patients in single-center cohort studies using the consensus definition [6, 7]. The prognosis of RAS and mixed phenotype are worse compared to the BOS phenotype [6, 7].

So far, no prospective randomized trial has been performed in RAS and mixed phenotypes. In preparation of a multicenter drug trial, the aim of the current study was to calculate the course of pulmonary function assessed by spirometry and outcomes in these specific CLAD phenotypes. Surrogate endpoints for such a trial must be analyzed to allow sample size and power calculations. In most CLAD-studies forced expiratory volume in 1 second (FEV_1_) and forced vital capacity (FVC) change over time have been used as surrogate endpoints of disease progression. These endpoints may be flawed by missing data.

## Methods

A retrospective analysis was performed in bilateral LTx recipients with RAS and mixed phenotype, transplanted between 2009 and 2018 in five large European centers (Barcelona, Copenhagen, Foch, Hannover and Leuven) with follow-up spirometry. Decline in pulmonary function (PFT) was measured by FEV_1_ and FVC up to 12 months after fulfilling full diagnostic criteria of RAS or mixed phenotype as described [5]. Spirometry and body plethysmography were performed according to American Thoracic Society (ATS)/ European Respiratory Society (ERS) standards. Presence of persistent opacities on chest imaging with or without pleural changes and a loss of lung volume (total lung capacity < 90% baseline) were used for case definition [8, 9]. The mixed phenotype was defined by FEV1/FVC < 0.7, with accompanying total lung capacity (TLC) decline while RAS patients were purely restrictive on spirometry. Baseline FEV1 and FVC were defined as the measurements obtained at date of diagnosis of RAS or mixed phenotype.

This retrospective study was performed in accordance with the ethical guidelines of the 2000 Declaration of Helsinki and the standards of the 2008 Declaration of Istanbul. Anonymized data analysis in retrospective studies relying on measurements and therapies applied as part of routine care were covered by local ethics committee´s vote (No 2923–2015).

Standard treatment of CLAD consisted of azithromycin, montelukast and extracorporeal photopheresis depending on local availability and according to center praxis. All patients were on standard calcineurin-inhibitor based multidrug immunsusuppression including prednisolone, cell-cycle inhibitors and/or proliferation signal inhibitors.

With the aim to assess the feasibility of future therapeutic trials in patients with RAS/mixed patterns, we investigated different methods with their own surrogate endpoints to allow sample size and power calculations. Two different methods using imputation of missing values were applied. In the first method of imputation according to the EPOS trial (NCT02262299, [10]) in BOS, missing data due to graft loss were imputed as 50% of baseline values. For patients with missing data other than death and who have data at other time points, the worst of either the last observed PFT prior to the missing visit or the next observed PFT was imputed. In the second method, previously used in idiopathic pulmonary fibrosis (IPF) trials [11], missing values as a result of graft loss were assigned as zero (0). Missing data for other reasons than death were imputed with the average value from three patients with the smallest sum of squared differences at each visit with data that were not missing.

### Statistics

Data are presented as percentage or median with corresponding 25th and 75th percentiles. Data analysis was performed with SPSS ® Statistics 26 (IBM). All reported P values are two-sided, unless otherwise indicated. Little’s Test was used to test the null hypothesis that the missing data is missing completely at random. Median values were compared with the Student’s t test (in case of normality, linearity, and homoscedasticity), or the Mann–Whitney U test. Category variables were analysed using Chi^2^ test. Kaplan–Meier plots were used to illustrate the timing of events during follow-up and statistical assessment was performed by the log-rank test.

## Results

Seventy patients were identified (39 RAS and 31 mixed phenotype) in 5 centers. Hannover identified 24 patients, Barcelona 17, Leuven 9, Copenhagen 10 and Foch 10 patients. Patients demographics are displayed in Table 1. Twenty-nine (41%) were women, the median age was 54 years and patients were median 3.1 years after LTx at diagnosis of RAS/mixed phenotype.

**Table 1:** Patient demographics.

	(n = 70)
Gender, female–n (%)	29 (41)
Age at transplant (years)–median (25^th^, 75^th^ percentile)	51 (31, 57)
Diagnosis–n (%)	
cystic fibrosis	20 (29)
COPD	25 (36)
interstitial lung disease	14 (20)
other	11 (16)
Procedure type	
bilateral LTx	68 (97)
heart-lung transplantation	2 (3)
Time to CLAD (days)—median (25^th^, 75^th^ percentile)	962 (477, 1425)
Time from CLAD to RAS/mixed (days)—median (25^th^, 75^th^ percentile)	60 (0, 216)
Age at RAS, mixed phenotype (years)—median (25^th^, 75^th^ percentile)	54 (38, 60)
Re-do transplantation–n (%)	3 (4)
Deceased during follow-up–n (%)	50 (71)
Time from RAS to graft loss (days)—median (25^th^, 75^th^ percentile)	381 (247, 914)
FEV1% baseline—median (25^th^, 75^th^ percentile)	70 (55, 78)
FVC % baseline—median (25^th^, 75^th^ percentile)	73 (66, 85)
FEV1/FVC Ratio—median (25^th^, 75^th^ percentile)	0.71 (0.63, 0.80)
FEV1/FVC Ratio < 0.7 –n (%)	31 (44)
Graft survival—median days (95%-confidence interval)	895 (379, 1411)

FEV1 forced expiratory volume in one second, FVC–forced vital capacity, RAS–restrictive allograft syndrome, CLAD–chronic lung allograft dysfunction.

**Fig 1:**
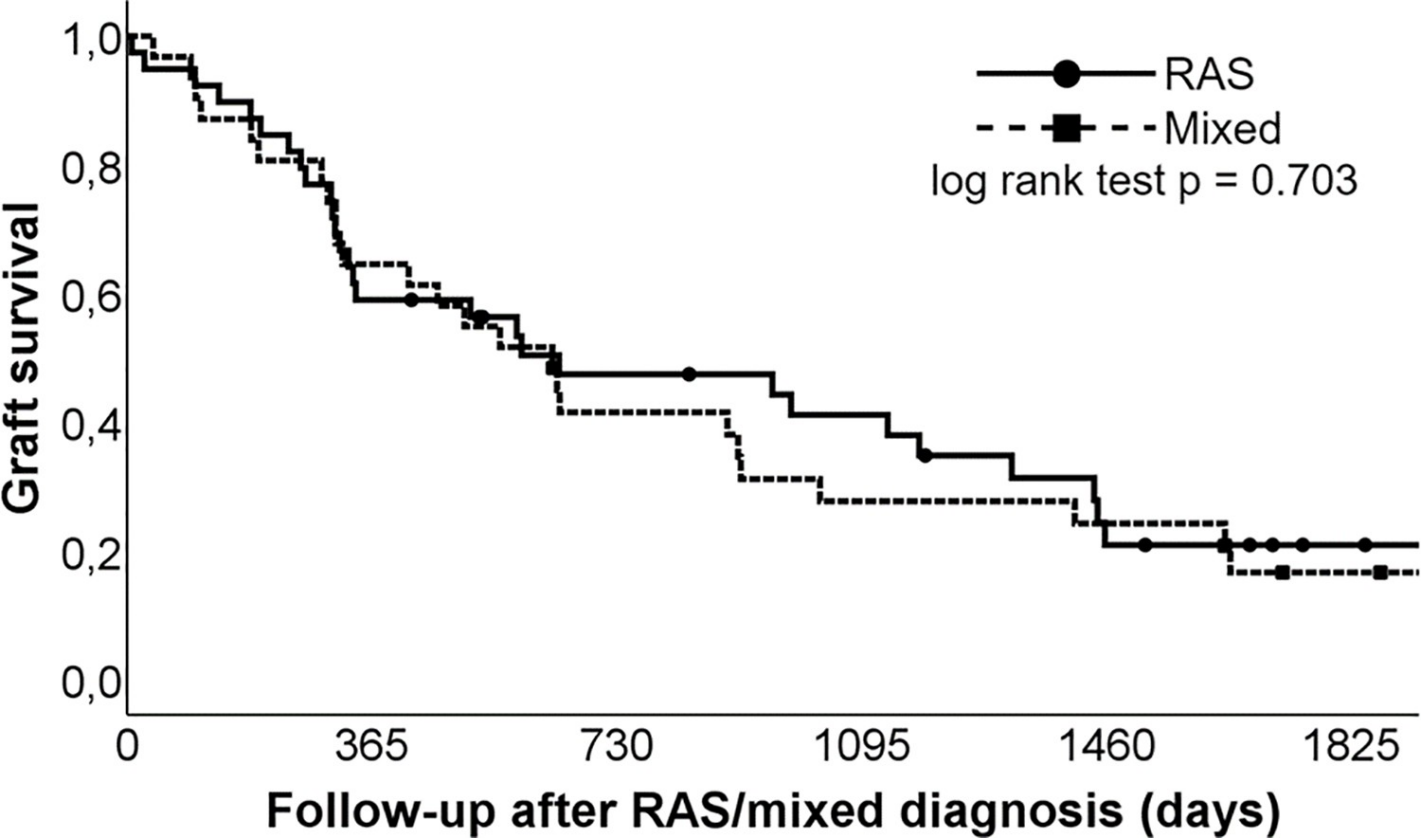
Kaplan-Meier curve of graft loss. RAS- restrictive allograft syndrome.

Eight, 13 and 25 patients died within 6, 9 and 12 months after RAS /mixed diagnosis, respectively. Three patients underwent re-transplantation (199, 259 and 463 days after diagnosis) leading to a post diagnosis graft survival of 89, 79 and 61% at 6, 9 and 12 months, respectively. Graft survival was not different between RAS and mixed phenotype (Fig 1).

PFT were missing in 10 (14%), 15 (21%) and 26 (37%) patients at 6, 9 and 12 months respectively with 96% of all missing values caused by death.

**Fig 2:**
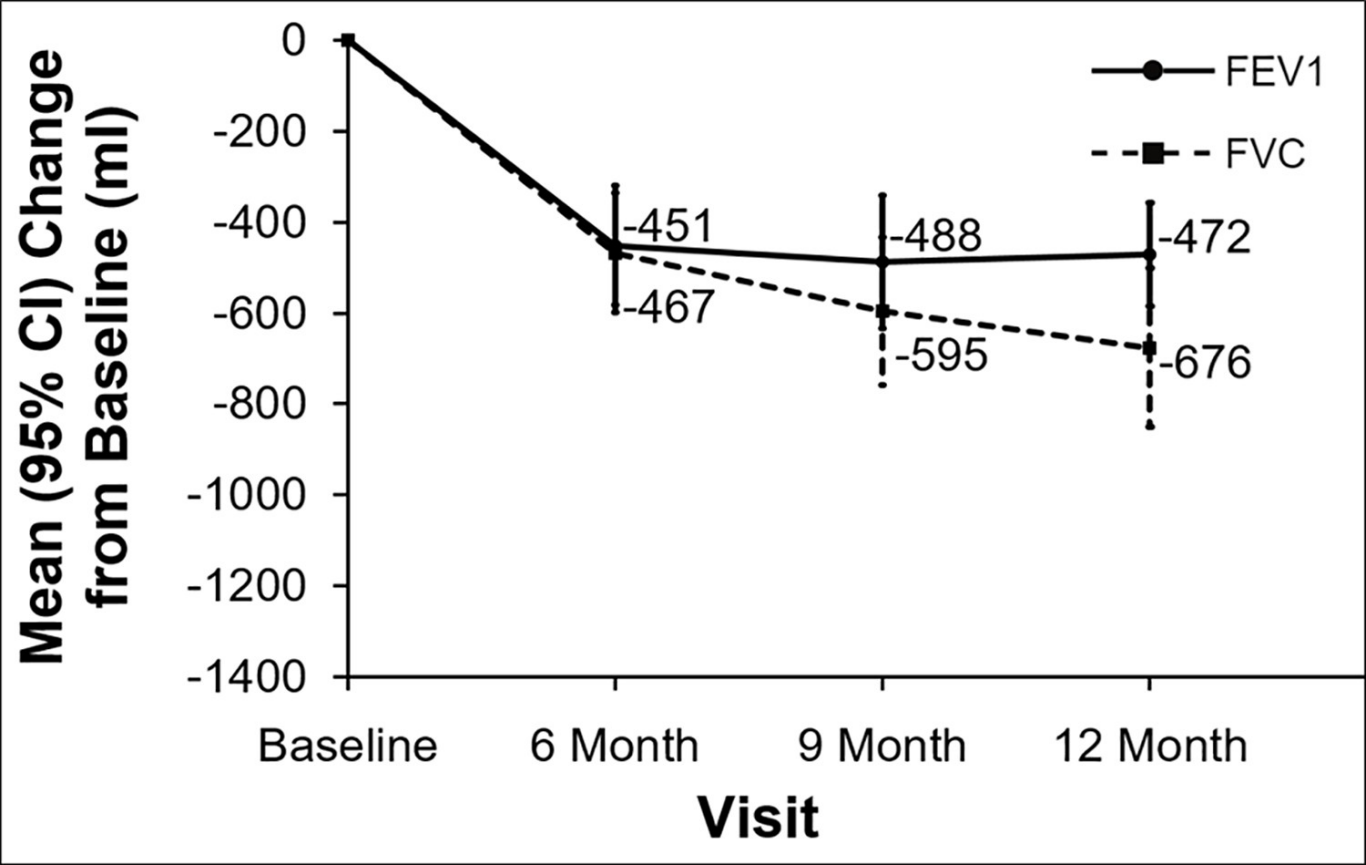
Pulmonary function during observation–values as observed. FEV_1_ –forced expiratory volume in 1 second, FVC- forced vital capacity, ml–milliliters, CI–confidence interval.

**Fig 3:**
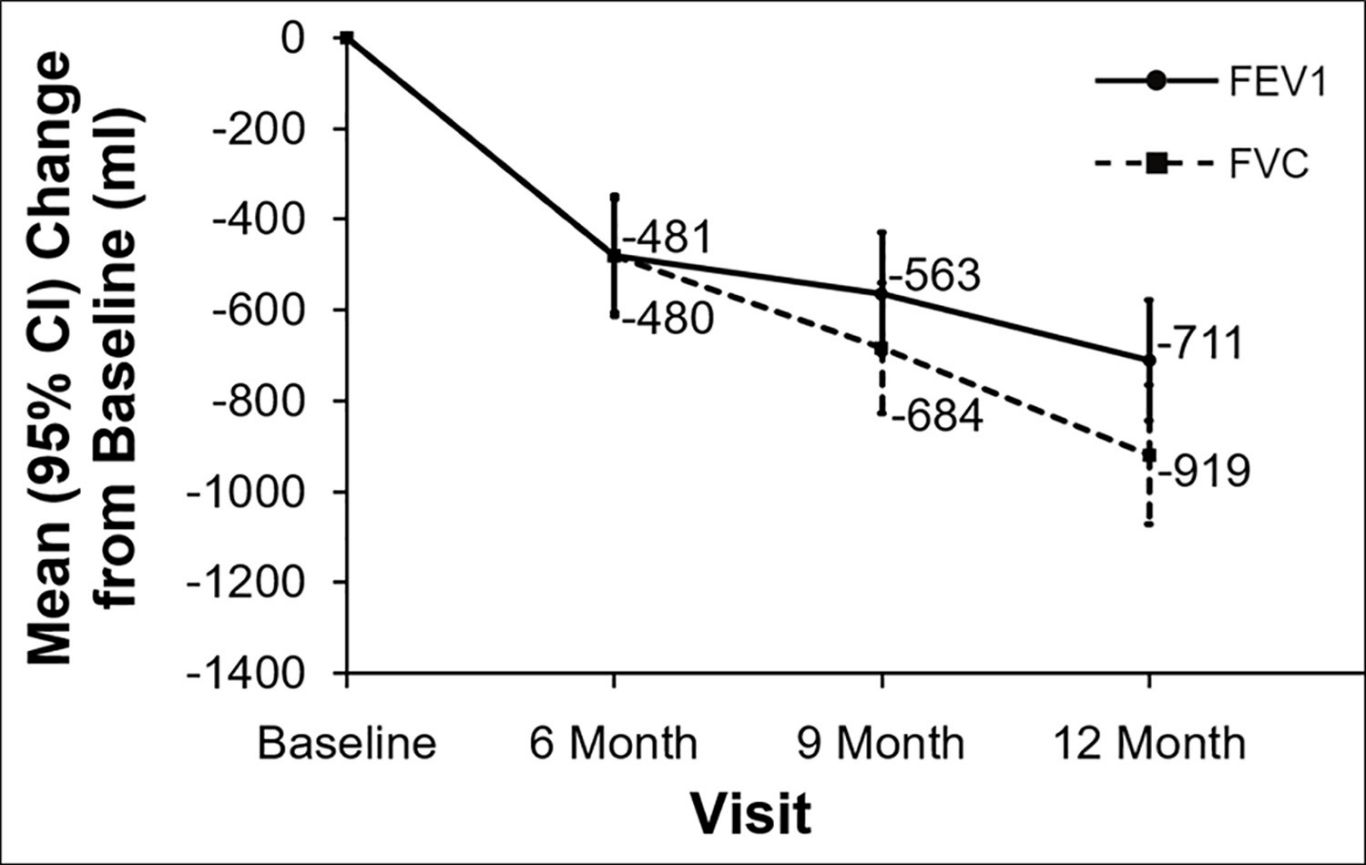
Pulmonary function during observation–imputation 50% baseline for death and worst of last or next observation for other reasons of missing values (method of imputation used in EPOS trial). FEV_1_ –forced expiratory volume in 1 second, FVC- forced vital capacity, ml–milliliters, CI–confidence interval.

**Fig 4:**
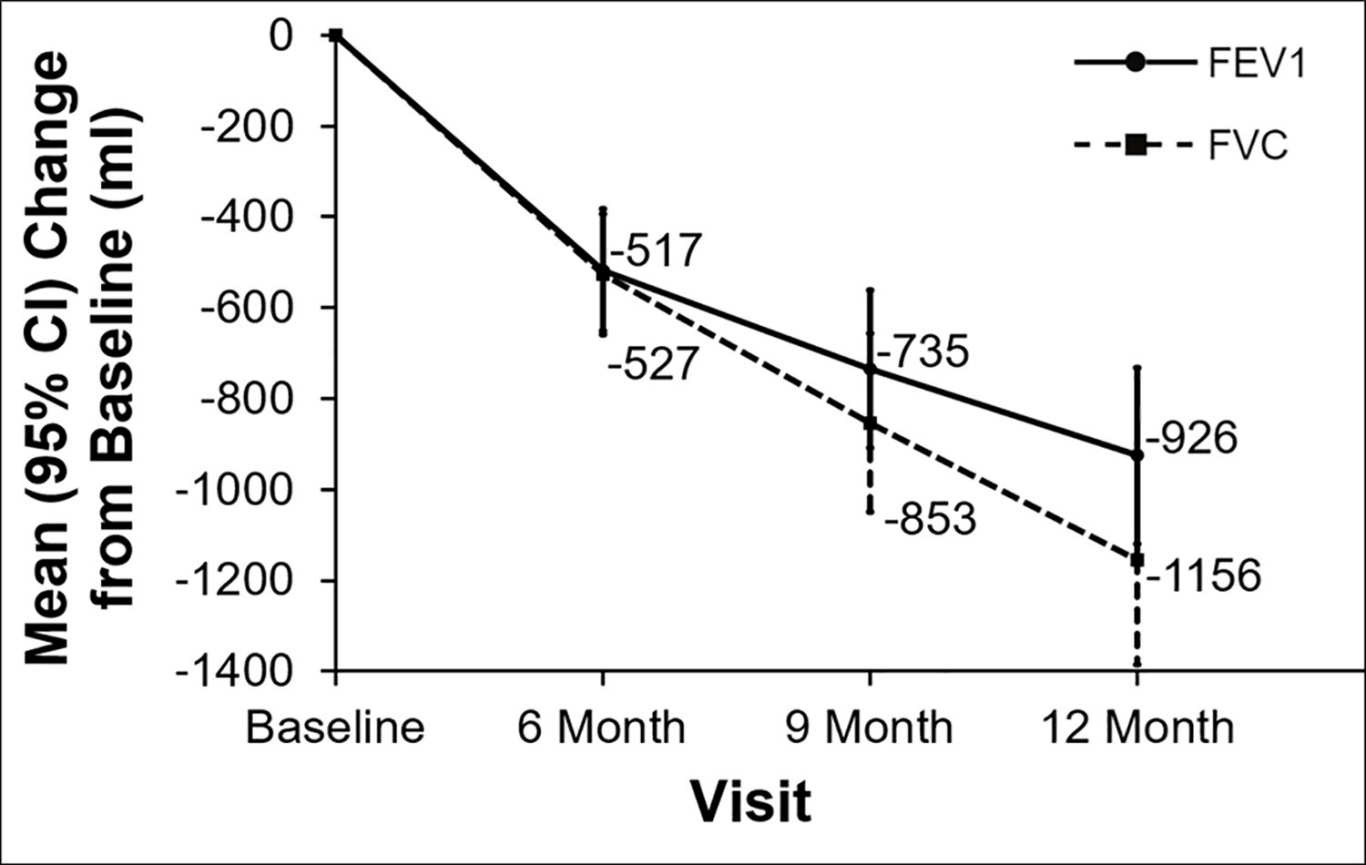
Pulmonary function during observation–PFT = 0 for death and sum of squared differences for other reasons of missing values method of imputation (used in CAPACITY trials). FEV_1_ –forced expiratory volume in 1 second, FVC- forced vital capacity, ml–milliliters, CI–confidence interval.

Observed loss of pulmonary function defined by mean FEV_1_ decline was 451 ml (Fig 2) at 6 months. Observed FEV1 stabilized at 9 and 12 months, while FVC showed continuous decline at both 9 and 12 months. Using the two different methods of imputation as described above (Fig 3 - method used in EPOS trial and Fig 4 - method used in CAPACITY trials) a progressive decline after 6 months for FEV1 was also noted. This decline was even more pronounced for FVC when using either of the imputation methods. The method used in the CAPACITY trials [11] with imputation of zero for missing values caused by death displayed a higher decline in both spirometric parameters in comparison to the imputation method used in the EPOS trial (Fig 3 and Fig 4). There was no change in FEV1/FVC ratio during follow-up. Values observed were median 0.72, 0.69, 0.70 and 0.71 at baseline and after 6, 9 and 12 months respectively.

## Discussion

To our knowledge, this is the largest follow up cohort of fully characterized patients with RAS or mixed CLAD phenotype according to the 2019 consensus definition. In this multicenter cohort, both phenotypes demonstrated persistent decline in graft function after imputation of missing values and a 36% mortality 12 months after diagnosis.

In a cohort of 25 recipients of RAS and mixed phenotype, the Toronto group reported a similar 1-year survival of 50% [6]. Two other single-center series reported survival in patients with RAS or mixed phenotype using various definitions. Todd et al. described 65 cases of CLAD patients with FEV1/FVC of 0.8 or above of whom 40 (77%) had opacities on imaging. In this cohort total lung capacity (TLC) was not used to identify loss of lung volume and a 40% 1-year survival was observed. Verleden et al. characterized 71 patients with persistent opacities, of whom 25 (35%) had obstructive physiology on PFT with a similar one year survival of 50% after CLAD-diagnosis. Unlike our study, FVC below 80% baseline was used as a surrogate of restrictive physiology in this study. Concurrent for these three studies, both RAS and mixed phenotype phenotypes seem to involve a high 1-year mortality. The use of TLC in the 2019 CLAD definition and the prerequisite of follow-up spirometry [12] made patients with rapid and severe deterioration (“white out” on imaging) unlikely to be included in our study which explains a slightly higher 1-year survival than expected from other publications.

As studies of RAS epidemiology were mostly done before the consensus definition was published in 2019, the term RAS used in historical studies may not have strictly met the current case definition and therefore these outcome data should not be used for planning a randomized clinical trial addressing these RAS patients.

Most trials in BOS have used FEV1 decline as the primary endpoint [13, 14] and the evolution of FVC has been used to characterize the natural course in RAS patients [15]. In historical observational studies of CLAD, a stabilization of FEV1 was frequently reported. Given the significant mortality of unselected CLAD patients, missing values are of great concern [4]. Furthermore patients with rapidly CLAD progression experience a worse prognosis where 58% died from respiratory failure, rather early after CLAD diagnosis [4]. Analyzing change in PFT as observed would therefore lead to a huge underestimation of disease progression in these patients [16]. Plotted FEV_1_ and FVC as observed in Fig 2 suggested a relative “stabilization” of FEV1 and a decline of FVC in our data. Observed loss of pulmonary function defined by mean FEV_1_ decline was almost double than reported in a randomized trial of BOS patients (451 compared to 255 ml) (3). In addition, imputation techniques demonstrated a further decline of both parameters.

In two large phase III randomized trials of antifibrotics in IPF the number of missing data was 13% and 15% during a follow up of 52 and 72 weeks with 48 to 66% of these cases attributed to death [11, 17]. In a recent early terminated trial studying pirfenidon in non-IPF progressive fibrotic interstitial lung diseases, 47% of spirometric values were missing with 10% attributed to death [18]. Values were missing overall in 24% of our cases and 96% were caused by patient death. Therefore the proportion of missings values can be transferred to a clinical study in CLAD patients. The limited number of missing values due to causes other than death reflects the meticulous follow-up of lung transplant reciepients in experienced centers and makes such a trial in CLAD patients with RAS or mixed phenotype feasible.

Rapid FEV1 decline is a well-known risk factor for death in CLAD [4]. Patients who died with CLAD are likely to have a faster FEV1 decline than those still alive at the end of a study.

Therefore, using FEV1 and FVC as outcome parameters of treatment responses in CLAD patients may lead to important misinterpretation of data. Given the high mortality of the specific phenotypes in the current study, survival might be a primary endpoint in clinical trials, but may increase patient numbers needed in clinical studies. The use of a composite end-point of death or FVC decline (e.g. 10% or more) is another way to deal with missing values owing to death. Unfortunately the natural FVC cut-off is unknown and dichotomisation of a metric variable like FEV1 or FVC leads to a considerable loss of statistical power. In our retrospectively analysis the proportion of patients with death or FVC decline of 10% or more was 61%.

In trials of nintedanib in IPF, the primary endpoint was the annual rate of decline in FVC [17]. This approach allowed for missing data by calculating mean FVC at each time-point over all nonmissing FVC values. Consequently, FVC is computed for a decreasing number of patients over time. This method implies that patients who dropped out had the same FVC decline as patients who did not and, as a consequence, the evolution of FVC is linear over time.

The last observation carried forward method assumes that there was no change in FVC value between the last point available and the missing time point. Imputation of 0 in case of graft loss makes the reasonable assumption that patients who died had worse FVC than patients who survived although this strategy greatly influences the estimation of mean decline in the whole population due to a huge loss in pulmonary function. In the EPOS trial, imputation of 50% of baseline spirometric results were used in case of missing values due to graft loss. This imputation might underestimate graft loss in patients with RAS and mixed phenotype due to the much faster decline in graft function in both these phenotypes. The imputation of zero might overestimate the effect of graft loss due to its large impact on graft function as an endpoint. The “sum of squared differences” method is a reasonable imputation for missing values according to our analysis.

The poor outcome in this series of specific phenotypes of CLAD patients suggests the urgent need for future therapeutic randomized trials, for instance anti-fibrotic agents. In comparison to IPF, the number of missing values in RAS/mixed phenotype seems to be even higher and most often attributed to death. Survival may be used as an endpoint in clinical trials with these distinct phenotypes. Imputation techniques are of relevance if graft function is used as a surrogate of disease progression in future trials.

## Supporting information

S1 File (PDF)

S1 Data (CSV)
