# Supplementary material for: Disease progression in patients with the restrictive and mixed phenotype of Chronic Lung Allograft dysfunction—A retrospective analysis in five European centers to assess the feasibility of a therapeutic trial
Source: PLoS One. 2021 Dec 23;16(12):e0260881. doi: 10.1371/journal.pone.0260881 (PMC8700042; doi:10.1371/journal.pone.0260881)
Supplement: S1 File — (PDF) [file pone.0260881.s001.pdf]

Medizinische Hochschule Hannover

Zentrum für Innere Medizin
Klinik für Pneumologie
Leitung: Prof. Dr. Tobias Welte

MHH-Klinik für Pneumologie, Carl-Neuberg-Straße 1, 30625 Hannover

Public Library of Science
Carlyle House
Carlyle Road
Cambridge CB4 3DN
United Kingdom

Lungentransplantation, OE6870
Prof. Dr.med. Jens Gottlieb
Telefon: 0511 532-3560
Fax: 0511 532-9094
gottlieb.jens@mh-hannover.de

Carl-Neuberg-Straße 1
30625 Hannover
Telefon: 0511 532-0
www.mh-hannover.de

Ihr Zeichen | Ihre Nachricht vom | Unser Zeichen

2021-05-17

Manuscript "Disease Progression in patients with the restrictive and mixed phenotype of Chronic Lung Allograft dysfunction – A Retrospective Analysis in five European Centers to assess the feasibility of a therapeutic trial"

Dear Professor Bellamy,

please find enclosed the ethics committee vote (original version 2015 and updated version of January 2021) concerning analyzing anonymized data form the data warehouse of the German Center of lung research (DZL) for retrospective studies. In addition this was approved by the federal data protection officer.

We hope that the manuscript will be of interest for the readership of your journal. For any further questions, please do not hesitate to contact me via e-mail at any time (gottlieb.jens@mh-hannover.de).

Sincerely Yours

Prof. Dr. Jens Gottlieb

Medizinische Hochschule Hannover

Ethikkommission
Vorsitzender:
Prof. Dr. Stefan Engeli

Sekretariat:

Marion Lange
Telefon: 0511 532-3443

Liane Höft
Telefon: 0511 532-9812

Fax: 0511 532-16 3443
ethikkommission@mh-hannover.de

Carl-Neuberg-Straße 1
30625 Hannover
Telefon: 0511 532-0
www.mh-hannover.de

MHH Ethikkommission OE 9515
30623 Hannover

Herrn
Prof. Dr. Thomas Illig
Leiter Biobank (HUB) – OE **9160**

22.01.2021/MLa

Nr. 2923-2015
Verwendung der DZL-weiten Patienten-Information und Einwilligungserklärung des Deutschen Zentrums für Lungenforschung (DZL) (Broad Consent)

Sehr geehrter Herr Kollege Illig,

als Vorsitzender der Ethikkommission habe ich Ihr Schreiben vom 21.01.2021 zur Kenntnis genommen. Die unten genannten Dokumente werden zur Kenntnis genommen. Gegen die zukünftige Verwendung dieser Dokumente bestehen keine Einwände.

- DZL-MHH-Broad Consent Patienteninformation, V2.0 vom 05.05.02020
- DZL-MHH-Broad Consent Patienteninformation - gesetzliche Betreuer, V2.0 vom 05.05.02020
- DZL-MHH-Broad Consent Probandeninformation, V2.0 vom 05.05.02020
- Ergänzendes Informationsschreiben für Studien-Teilnehmer gemäß Europäischer Datenschutz-Grundverordnung
- Weiteres Ergänzendes Informationsschreiben für Studien-Teilnehmer gemäß Europäischer Datenschutz-Grundverordnung - Übermittlung in Drittländer

Die Ethikkommission weist darauf hin, dass die ärztliche und juristische Verantwortung bei den jeweiligen Prüfärzten verbleibt.

Datenschutzrechtliche Aspekte von Forschungsvorhaben werden durch die Ethikkommission grundsätzlich nur kursorisch geprüft. Dieses Votum / diese Bewertung ersetzt mithin nicht die Konsultation des zuständigen Datenschutzbeauftragten.

Mit besten Grüßen

Prof. Dr. Stefan Engeli
Vorsitzender der Ethikkommission

Nachrichtlich:
Bernemann.Inga@mh-hannover.de

Medizinische Hochschule Hannover

Ethikkommission
Vorsitzender:
Prof. Dr. H. D. Tröger

Sekretariat:
Rita Landowski
Telefon: 0511 532-3443
Fax: 0511 532-5423
ethikkommission@mh-hannover.de

Carl-Neuberg-Straße 1
30625 Hannover
Telefon: 0511 532-0
www.mh-hannover.de

MHH Ethikkommission OE 9515
30623 Hannover

Herrn
Prof. Dr. Thomas Illig
Hannover Unified Biobank
9160 im Hause

04.11.15/La

Verwendung der DZL-weiten Patienten-Information und Einwilligungserklärung des Deutschen Zentrums für Lungenforschung (DZL) (Broad Consent)
Nr. 2923-2015

Sehr geehrter Herr Kollege Illig,

als Vorsitzender habe ich im Auftrag der Mitglieder der Ethikkommission Ihr Schreiben vom 02.11.15 geprüft. Es bestehen keine ethisch-rechtlichen Bedenken gegenüber der Verwendung der DZL-Patienteninformation und Einwilligungserklärung am Standort Hannover.

Mit besten Grüßen bin ich
Ihr

H. D. Tröger

Prof. Dr. H. D. Tröger
Vorsitzender

PATIENTENINFORMATION

(BREATH/Version 1.0 vom 23.09.2014)

Sehr geehrte Patientin, sehr geehrter Patient!

Sie werden gegenwärtig als Patient* der Medizinischen Hochschule Hannover (MHH) ärztlich behandelt.
Unsere Einrichtung ist Mitglied im Deutschen Zentrum für Lungenforschung (DZL), einer vom Bundesministerium für Bildung und Forschung unterstützten Einrichtung, die sich die weitere Erforschung und die Entwicklung neuer Therapieverfahren von und für Lungenerkrankungen zum Ziel gemacht hat. Als Teil dieser Bemühungen wird das DZL Patientendaten und Biomaterialien sammeln, zusammenführen und zentral verwalten, um hierüber die wissenschaftliche Erforschung zu ermöglichen. Die im DZL vereinten Biomaterialien werden in lokalen Biobanken, im konkreten Fall der Hannover Unified Biobank (HUB), aufbewahrt. Gesammelt werden menschliche Biomaterialien wie z.B. Blut, Gewebe oder bronchoalveoläre Lavagen oder Sputum, verknüpft mit ausgewählten medizinischen Daten.

Die Untersuchung von menschlichen Biomaterialien und die Analyse der daraus gewonnenen oder zu gewinnenden Daten sind zu einem wichtigen Instrument medizinischer Forschung geworden. Deshalb fragen wir unsere Patienten und daher auch Sie, ob Sie bereit sind, uns bestimmte Körpermaterialien und Daten für die Forschung zur Verfügung zu stellen. Ihre Teilnahme ist völlig freiwillig. Soweit Sie sich nicht beteiligen möchten oder Ihre Zustimmung später widerrufen möchten, erwachsen Ihnen daraus keine Nachteile.

Im Folgenden informieren wir Sie über die Ziele der Hannover Unified Biobank (HUB), welche eine Einrichtung der MHH und Mitglied in der Plattform Biobanking des DZLs am Standort BREATH ist, sowie über die Verfahrensweisen und die Maßnahmen zum Schutz Ihrer personenbezogenen Daten, damit Sie sich auf dieser Grundlage Ihre eigene Meinung bilden und eine Entscheidung treffen können.

Sollte Ihnen etwas unklar sein, fragen Sie bitte Ihren behandelnden Arzt bzw. Ihren Studienarzt, bevor Sie Ihre Zustimmung erteilen. Sie können sich wegen Rückfragen auch zu einem späteren Zeitpunkt an Prof. Dr. med. Jens Gottlieb wenden.

Informationen zur Bronchoskopie

Die Bronchoskopie gehört zu den Standardverfahren zur Früherkennung von Problemen nach Transplantation und dient auch der Überwachung der Organfunktion. Sie ist Ihnen als Routinemaßnahme nach Lungentransplantation bereits vertraut. Die Lunge als transplantiertes Organ kann dabei direkt angeschaut werden, dies ist beispielsweise bei anderen Transplantationsverfahren (z.B. Niere, Leber, Herz) nicht direkt möglich.

Bei einer Bronchoskopie wird ein optisches Instrument (Bronchoskop) durch die Nase oder den Mund in die Luftröhre eingeführt und in die Bronchien vorgeschoben. Sie erhalten vorher eine örtliche Betäubung mit einem Spray, so dass die Untersuchung nicht schmerzhaft ist. Die Atemfunktion wird sehr genau überwacht und sicherheitshalber Sauerstoff gegeben. Bei Lungentransplantierten ist eine Beruhigungsspritze nur ganz selten erforderlich. Durch die Untersuchung lassen sich die Atemwege und das transplantierte Organ gut überblicken und

* Im Rahmen dieses Textes schließt die männliche Bezeichnung stets die weibliche Bezeichnung mit ein.

es können mit kleinsten Instrumenten Gewebsteile, Schleim- oder Spülmaterial zur feingeweblichen und bakteriologischen Untersuchung entnommen werden.
Auch bei sorgfältigster Untersuchung können mögliche Komplikationen nicht ausgeschlossen werden. Komplikationen sind aber insgesamt sehr selten (weniger als 1%). Komplikationen können sein Verletzungen des Kehlkopfes, der Luftröhre und der Bronchien, Blutungen, Schluckbeschwerden, Schmerzen, leichte Heiserkeit, ein Durchbruch (Perforation mit der Folge einer Mittelfellentzündung), Verschlechterung der Stimme, allergische Reaktionen auf örtliches Betäubungsmittel, Blutergüsse, Stimmbandschwellungen, Atemnot, Infektionen, Blutvergiftung (Sepsis), Herzinnenhautentzündung (Endokarditis), Stimmritzenkrampf (Laryngospasmus), Haut-, Weichteil- und Nervenschäden, Herzrhythmusstörungen, Gebissschäden, Druckschäden an Nerven, Gelenke oder der Wirbelsäule, langandauernde Beschwerden (z.B. Narben, Taubheitsgefühl), Bindehautreizung. Extrem selten (unter 0,1%) sind Atemversagen bis hin zum Atemstillstand und schwere Herz-Kreislaufkomplikationen.

Lufteintritt in den Rippenfellraum (Pneumothorax) ist nach transbronchialer Biopsie in einer Größenordnung von ca. 1% zu erwarten und muss nur ganz selten mit einem Drainageschlauch behandelt werden. Die Mehrzahl der Fälle heilt nach kurzer Überwachung von selbst ab. Blutung nach transbronchialer Biopsie sind mit etwa 5% zu erwarten, fast immer sind die Blutungen mit dem Bronchoskop in gleicher Sitzung zu stillen und die Patienten müssen nur ganz selten (<0,1%) stationär aufgenommen oder gar beatmet werden.

12 Stunden vor der Untersuchung sollten Sie nichts essen und nichts trinken. Die Immunsuppressiva (Sandimmun oder Prograf) sollten sie aber mit einem Schluck Wasser vor der Untersuchung einnehmen. Eine kurzfristige Heiserkeit sowie leichtere Blutspuren im Auswurf während der ersten 2 Tage nach der Untersuchung brauchen Sie nicht zu beunruhigen. Etwa 15 % der Patienten haben nach einer bronchialen Spülung (BAL) vorübergehendes (d.h. am Abend nach der Untersuchung) Fieber. Wenn dies auch am Folgetag der Untersuchung noch fortbesteht, sollten Sie das Transplantationszentrum benachrichtigen. Bitte nehmen Sie wegen der vorausgegangenen Betäubungsmittel 2 Stunden nach der Untersuchung keine Flüssigkeit oder Nahrung ein. Bitte verlassen Sie das Krankenhaus nach der Untersuchung nur nach Rücksprache mit dem Personal. Nach Biopsie ist eine Kontroll-Röntgenuntersuchung zum Ausschluss eines Lungenrisses notwendig. Nach Gabe von Beruhigungsmitteln dürfen Sie mindestens 24 Stunden kein Kraftfahrzeug steuern und keine Maschinen bedienen.

1. Ziele der Biobank

Die Hannover Unified Biobank (HUB) der MHH als Mitglied der DZL-Biobank, dient der Förderung medizinischer Forschung. In der Biobank werden Biomaterialien und ausgewählte medizinische Daten langfristig aufbewahrt und für die Erforschung von Lungenerkrankungen zur Verfügung gestellt, um die Vorbeugung, Erkennung und Behandlung von Lungenerkrankungen zu verbessern.

2. Um welche Art von Biomaterialien und Daten handelt es sich?

In der Hannover Unified Biobank (HUB), der lokalen DZL-Biobank, werden Biomaterialien wie Blut, Lungengewebe (Biopsien sowie chirurgisch entnommenes Lungengewebe), Sputum, bronchoalveoläre Lavagen, Spülflüssigkeit der Atemwege, exhalative Atemkondensate, Urin, Zellen, Abstriche und Luftproben gesammelt.
Bei dem Biomaterial handelt es sich um Gewebe und Körperflüssigkeiten, die im Laufe Ihres derzeitigen oder zukünftigen Krankenhausaufenthaltes zum Zweck der Untersuchung /

Behandlung entnommen, dafür jedoch nicht mehr benötigt werden und daher ansonsten vernichtet würden.
Die erhobenen Daten umfassen sowohl medizinische Daten (z.B. Alter, Geschlecht, verordnete Medikamente, familiäre Vorbelastung für bestimmte Erkrankungen, bekannte genetische Veränderungen) als auch Untersuchungsbefunde ihres derzeitigen oder zukünftigen Krankenhausaufenthaltes (z.B. Röntgenbilder, Ultraschallbefunde, Laborwerte, Lungenfunktionen).

Das DZL strebt grundsätzlich an, an mehreren Zeitpunkten die oben erwähnten Biomaterialien und begleitende medizinischen Daten zu erheben. Wir werden daher auch bei späterer Vorstellung in unserem Haus die o.g. Proben und erhobenen medizinischen Daten sammeln.

3. Wie werden die Biomaterialien und Daten verwendet?
Die von Ihnen zur Verfügung gestellten Biomaterialien und Daten werden ausschließlich für die medizinische Forschung bereitgestellt. Sie sollen im Sinne eines breiten Nutzens für die Allgemeinheit für viele verschiedene medizinische Forschungszwecke verwendet werden.

Aus einem kleinen Stück Ihres Restgewebes (z.B. nach Operation bzw. endoskopischer Biopsie und histologischer Begutachtung) oder aus Ihrem Blut, sollen nach Eignungsprüfung gegebenenfalls auch Gewebe und/oder Zellkulturen angezüchtet werden. Nach erfolgreicher Kultivierung sollen verschiedene Zellfunktionen wie Wachstum und Widerstandsfähigkeit sowie die Bildung wichtiger Proteine untersucht werden, um deren Bedeutung für die Steuerung verschiedener Zellfunktionen besser zu verstehen.
Die angezüchteten Zellen sollen für spätere Versuche gelagert werden, um auch zukünftige Fragestellungen beantworten zu können. Ziel aller Untersuchungen ist es, ein besseres Verständnis über molekularbiologische Vorgänge bei Entstehung, Wachstum und Ausbreitung von Lungenerkrankungen zu erreichen und ggf. neue Therapieansätze zu finden.

Zum derzeitigen Zeitpunkt können noch nicht alle zukünftigen medizinischen Forschungsziele beschrieben werden. Diese können sich sowohl auf Lungenerkrankungen als auch auf heute zum Teil noch unbekannte Krankheiten und genetische Defekte, auch anderer Organsysteme, beziehen. Es kann also sein, dass Ihre Proben und Daten auch für medizinische Forschungsfragen verwendet werden, die wir heute noch nicht absehen können. Deshalb werden an Ihren Biomaterialien möglicherweise auch genetische Untersuchungen, also Untersuchungen der Erbsubstanz, durchgeführt, und zwar unter Umständen auch eine Untersuchung Ihres gesamten Genoms.
Die Biomaterialien und Daten sollen für unbestimmte Zeit aufbewahrt und für die medizinische Forschung bereitgestellt werden.

Wenn Sie mit der oben beschriebenen Art und Dauer der Nutzung nicht in vollem Umfang einverstanden sind, werden Ihre Biomaterialien und Daten nicht in die Hannover Unified Biobank (HUB) der MHH aufgenommen.

4. Welche Risiken sind mit Ihrer Spende verbunden?
a. Gesundheitliche Risiken:
Da wir für die Biobank von Patienten lediglich Körpermaterial verwenden wollen, das im Rahmen der bei Ihnen vorgesehenen diagnostischen oder therapeutischen Maßnahmen

ohnehin entnommen wird und als Restmaterial normalerweise vernichtet würde, ist die Spende für Sie mit keinem zusätzlichen gesundheitlichen Risiko verbunden.
Falls im Rahmen einer Routine-Blutentnahme *zusätzlich* bis zu 25ml Blut abgenommen wird (das entspricht etwa 5 Teelöffeln), ist diese Entnahme für Sie mit keinem zusätzlichen gesundheitlichen Risiko verbunden.

b. Weitere Risiken:
Bei jeder Erhebung, Speicherung und Übermittlung von Daten aus Ihren Biomaterialien im Rahmen von Forschungsprojekten bestehen Vertraulichkeitsrisiken (z.B. die Möglichkeit, Sie zu identifizieren), insbesondere im Hinblick auf die Information zu Ihrer Erbsubstanz. Diese Risiken lassen sich nicht völlig ausschließen und steigen, je mehr Daten miteinander verknüpft werden können, insbesondere auch dann, wenn Sie selbst (z.B. zur Ahnenforschung) genetische Daten im Internet veröffentlichen. Wir weisen ferner darauf hin, dass für genetische Daten in Deutschland kein Beschlagnahmeverbot besteht. Zur Aufdeckung einer schweren Straftat darf die deutsche Polizei mit richterlichem Beschluss Einsicht in wissenschaftliche Daten verlangen.

Die Hannover Unified Biobank (HUB) der MHH versichert Ihnen, alles nach dem Stand der Technik Mögliche zum Schutz Ihrer Privatsphäre zu tun und Proben und Daten nur an Projekte weitergeben, die ein geeignetes Datenschutzkonzept vorweisen können (siehe Punkt 8: „Wer hat Zugang zu Ihren Biomaterialien und Daten?“).

5. Welcher Nutzen ergibt sich für Sie persönlich?
Persönlich können Sie für Ihre Gesundheit keinen unmittelbaren Vorteil oder Nutzen aus der Spende Ihrer Proben und Daten erwarten. Die Ergebnisse sind ausschließlich zu Forschungszwecken bestimmt.

Eine Rückmeldung von Ergebnissen aus der Untersuchung der Biomaterialien ist in der Regel nicht vorgesehen. Eine Rückmeldung kann nur dann erfolgen, wenn eine Information für Ihre Gesundheit aus unserer Sicht von erheblicher Bedeutung ist. Dies ist dann der Fall, wenn der Ausbruch einer (ggf. lebensbedrohlichen) Krankheit vermieden oder eine bisher möglicherweise nicht erkannte Krankheit behandelt werden kann.

6. Welcher Nutzen ergibt sich für unsere Gesellschaft?
Alle derzeit durchgeführten wie auch künftige medizinisch-wissenschaftliche Forschungsvorhaben zielen auf eine Verbesserung unseres Verständnisses für die Krankheitsentstehung und die Diagnosestellung und auf dieser Basis auf die Neuentwicklung von verbesserten Behandlungsansätzen. Informationen über die Aktivitäten der Biobank finden Sie unter www.dzl.de.

7. Welche Voraussetzungen gelten für die Nutzung Ihrer Biomaterialien und Daten?
a. Voraussetzung für die Gewinnung und Nutzung Ihrer Biomaterialien samt den zugehörigen personenbezogenen Daten zu Forschungszwecken ist Ihre schriftliche Einwilligung. Ihre Einwilligung ist freiwillig und kann jederzeit widerrufen werden (siehe auch Punkt 11 „Was beinhaltet Ihr Widerrufsrecht?“).

b. Ihre Biomaterialien und Daten werden in der Hannover Unified Biobank (HUB) der MHH, der lokalen DZL-Biobank des Standortes BREATH unter standardisierten Qualitäts- und Sicherheitsbedingungen unbefristet aufbewahrt und auf Antrag für Forschungszwecke herausgegeben. Sie werden nach dem Stand der Technik vor unbefugtem Zugriff gesichert.

c. Voraussetzung für die Verwendung der Biomaterialien und Daten für ein konkretes medizinisches Forschungsprojekt innerhalb des DZL ist, dass das Forschungsvorhaben durch eine Ethikkommission bewertet wurde. Die Ethikkommission prüft die ethischen und rechtlichen Aspekte des Forschungsprojekts.

8. Wer hat Zugang zu Ihren Biomaterialien und Daten und wie werden sie geschützt?

a. Alle unmittelbar Ihre Person identifizierenden Daten (Name, Geburtsdatum, Anschrift etc.) werden unverzüglich nach ihrer Gewinnung durch einen Code ersetzt (pseudonymisiert, verschlüsselt). Danach wird der Datensatz nochmals neu kodiert und gespeichert. Diese doppelte Kodierung schließt eine Identifizierung Ihrer Person durch Unbefugte nach heutiger Kenntnis weitestgehend aus. Erst in dieser Form werden die Biomaterialien und Daten für Forschungszwecke zur Verfügung gestellt.

b. Die Sie unmittelbar identifizierenden Daten bleiben in dem Krankenhaus, in dem die Proben und Daten gewonnen wurden und werden dort getrennt von den Biomaterialien und medizinischen Daten gespeichert. Ein Zugriff auf sie erfolgt nur, um entweder weitere oder fehlende medizinische Daten aus Ihren Krankenunterlagen zu ergänzen oder erneut mit Ihnen in Kontakt zu treten, falls Sie der Kontaktaufnahme zugestimmt haben (s. unten, Punkt 10). Eine Weitergabe der personenbezogenen Daten an Forscher oder andere unberechtigte Dritte, etwa Versicherungsunternehmen oder Arbeitgeber, erfolgt nicht.

c. Die doppelt verschlüsselten Biomaterialien und medizinischen Daten können auf Antrag, für medizinische Forschungszwecke nach zuvor festgelegten Kriterien an andere Universitäten, Forschungsinstitute und forschende Unternehmen, ggf. auch im Ausland weitergegeben werden. Dabei werden die Daten unter Umständen auch mit medizinischen Datensätzen in anderen Datenbanken verknüpft, sofern die gesetzlichen Voraussetzungen hierfür erfüllt sind.

d. Biomaterialien und Daten, die an Dritte weitergegeben wurden, dürfen nur für den beantragten Forschungszweck verwendet und vom Empfänger nicht zu anderen Zwecken weitergegeben werden. Nicht verbrauchtes Material wird an die Biobank zurückgegeben oder vernichtet.

e. Wissenschaftliche Veröffentlichungen von Ergebnissen erfolgen ausschließlich anonymisiert, also in einer Form, die keine Rückschlüsse auf Ihre Person zulässt.

9. Erlangen Sie einen finanziellen Vorteil aus der Nutzung Ihrer Biomaterialien und Daten?

Für die Überlassung Ihrer Biomaterialien und Daten erhalten Sie kein Entgelt. Sollte aus der Forschung ein kommerzieller Nutzen erzielt werden, werden Sie daran nicht beteiligt.
Mit der Überlassung der Biomaterialien an die Medizinische Hochschule Hannover (MHH), die lokal die DZL-Biobank vertritt, werden diese Eigentum der MHH. Ferner ermächtigen Sie die MHH, Ihre Daten zu nutzen. Dabei haben Sie das Recht, fehlerhaft gespeicherte oder bearbeitete Daten jederzeit zu berichtigen.

Für die Bereitstellung der Proben und Daten kann die Biobank von den Nutzern eine Aufwandsentschädigung erheben.

10. Erfolgt eine erneute Kontaktaufnahme mit Ihnen?

Zur Erhebung von weiteren Verlaufsdaten kann es sinnvoll werden, zu einem späteren Zeitpunkt erneut Kontakt mit Ihnen aufzunehmen, um ergänzende Informationen und/oder Biomaterialien von Ihnen zu erbitten. Zudem kann die erneute Kontaktaufnahme genutzt werden, um z. B. Ihre Einwilligung zum Abgleich mit anderen Datenbanken einzuholen oder Ihnen /Ihrem behandelnden Arzt/Studienarzt/ Ihrem Hausarzt eine Rückmeldung über für Sie gesundheitlich relevante Ergebnisse zu geben (s. oben Punkt 5).

Sollte eine solche Kontaktaufnahme von Ihnen erwünscht sein, wird dies durch unsere Klinik erfolgen.
Falls Sie eine erneute Kontaktaufnahme nicht wünschen, kreuzen Sie bitte das entsprechende Kästchen in der Einwilligungserklärung an.

11. Was beinhaltet Ihr Widerrufsrecht?

Sie können Ihre Einwilligung zur Verwendung Ihrer Biomaterialien und Daten jederzeit ohne Angabe von Gründen und ohne nachteilige Folgen für Sie widerrufen. Im Falle eines Widerrufs können Sie entscheiden, ob Ihre Biomaterialien vernichtet und die dazu gehörenden Daten gelöscht werden sollen, oder ob sie in anonymisierter Form für weitere Forschungsvorhaben verwendet werden dürfen. Sobald der Bezug der Biomaterialien und der übrigen Daten zu Ihrer Person gelöscht wurde, ist eine Vernichtung jedoch nicht mehr möglich. Zudem können Daten aus bereits durchgeführten Analysen nicht mehr entfernt werden. Trotz Widerrufs kann eine spätere Zuordnung des genetischen Materials zu Ihrer Person über andere Quellen niemals ausgeschlossen werden.

Wenden Sie sich für einen Widerruf bitte an:

Prof. Dr. med. Jens Gottlieb
Klinik für Pneumologie – OE6870
Medizinische Hochschule Hannover
Carl-Neuberg-Straße 1
30625 Hannover

EINWILLIGUNGSERKLÄRUNG

Name:	Vorname:	Geburtsdatum:

Ich bin damit einverstanden, dass meine Biomaterialien und Daten, wie in der Patienteninformation beschrieben, an die Hannover Unified Biobank (HUB) der Medizinischen Hochschule Hannover, der lokalen DZL-Biobank am Standort BREATH, gegeben und für medizinische Forschungszwecke verwendet werden.
Dies betrifft unter Umständen auch die Weitergabe für Forschungsprojekte im Ausland mit möglicherweise niedrigerem Datenschutzniveau. Das Eigentum an den Biomaterialien übertrage ich an die lokal mit der Biomaterial-Sammlung beauftragte Hannover Unified Biobank (HUB) der Medizinischen Hochschule Hannover am DZL-Standort BREATH**.**

Ich habe die Patienteninformation gelesen und hatte die Gelegenheit, Fragen zu stellen.

Ich weiß, dass meine Teilnahme freiwillig ist und ich meine Einwilligung jederzeit ohne Angabe von Gründen widerrufen kann, ohne dass mir daraus irgendwelche Nachteile entstehen.

Ich bin damit einverstanden, dass ich bzw. mein Hausarzt evtl. zu einem späteren Zeitpunkt erneut kontaktiert wird (falls nicht gewünscht, bitte „nein“ ankreuzen).

- zum Zweck der Gewinnung weiterer Informationen / Biomaterialien, □ **ja** □ **nein**
- zum Zweck der Einwilligung in den Abgleich mit anderen Datenbanken, □ **ja** □ **nein**
- zum Zweck der Rückmeldung für mich gesundheitsrelevanter Ergebnisse □ **ja** □ **nein**

Diese Rückmeldung soll erfolgen über die Einrichtung, in der meine Biomaterialien / Daten gewonnen wurden oder über folgenden Arzt (falls gewünscht, bitte angeben)

Name und Anschrift des Arztes: ....

DATENSCHUTZERKLÄRUNG

Ich erkläre mich damit einverstanden, dass die Medizinische Hochschule Hannover (MHH), wie in der Patienteninformation beschrieben,

- personenbezogene Daten von mir erhebt und speichert,
- weitere Angaben über meine Gesundheit aus meinen Krankenunterlagen entnimmt,
- und die Daten gemeinsam mit meinen Biomaterialien pseudonymisiert (das heißt verschlüsselt) der DZL-Biobank für medizinische Forschungsvorhaben zur Verfügung stellt.

Die Biomaterialien und Daten dürfen unbefristet für medizinische Forschungsvorhaben verwendet werden.

Sie dürfen pseudonymisiert nach Prüfung und Beratung durch die zuständige öffentlich rechtliche Ethikkommission an Universitäten, Forschungsinstitute und forschende Unternehmen, ggf. auch im Ausland, zu Zwecken medizinischer Forschung weitergegeben werden.

Ich bin darüber aufgeklärt worden, dass ich meine Einwilligung gegenüber der DZL-Biobank ohne Angabe von Gründen jederzeit widerrufen kann. Beim Widerruf werden auf mein Verlangen die verbliebenen Biomaterialien und die erhobenen Daten vernichtet bzw. gelöscht oder anonymisiert. Daten aus bereits durchgeführten Analysen können nicht mehr entfernt werden.

Eine Kopie der Patienteninformation und Einwilligungserklärung habe ich erhalten. Das Original verbleibt bei der Medizinischen Hochschule Hannover (MHH).

____

Name des Patienten *(in Druckbuchstaben)*

________________ ____

Ort, Datum *(vom Patienten einzutragen)* und Unterschrift des Patienten

Ich habe das Aufklärungsgespräch geführt und die Einwilligung des Patienten eingeholt.

____

Name des Arztes *(in Druckbuchstaben)*

________________ ____________________

Ort, Datum und Unterschrift des Arztes

PATIENTENINFORMATION

(Version 2.0 vom 29.06.2020)

Patienten-Etikett

Sehr geehrte Patientin, sehr geehrter Patient!

Sie werden gegenwärtig als Patient* der Medizinischen Hochschule Hannover (MHH) ärztlich behandelt.

Unsere Einrichtung ist Mitglied im Deutschen Zentrum für Lungenforschung (DZL), einer vom Bundesministerium für Bildung und Forschung unterstützten Einrichtung, die sich die weitere Erforschung und die Entwicklung neuer Therapieverfahren von und für Lungenerkrankungen zum Ziel gemacht hat. Als Teil dieser Bemühungen werden bestimmte Körpermaterialien wie z.B. Blut, Urin, Gewebe, Sputum oder bronchoalveoläre Lavagen in einer sogenannten Biobank, der Hannover Unified Biobank (HUB), gesammelt. Ausgewählte, und mit den Proben verknüpfte, medizinische Daten werden in einer separaten Datenbank gespeichert.

In einem zweiten Schritt werden die lokal gespeicherten Daten in die DZL Datenbank sogenanntes DZL-Data Warehouse (http://www.dzl.de/index.php/de/forschung/plattformen/biobank) überführt, und mit den Biomaterialien zusammen zentral verwaltet und ausgewertet. Dabei werden die lokal in der HUB-Biobank gelagerten Biomaterialien auf Anfrage der DZL-Biobank für wissenschaftliche Untersuchungen weitergegeben.

Die Untersuchung von menschlichen Biomaterialien und die Analyse der daraus gewonnenen oder zu gewinnenden Daten sind zu einem wichtigen Instrument medizinischer Forschung geworden. **Deshalb fragen wir unsere Patienten und daher auch Sie, ob Sie bereit sind, uns bestimmte Körpermaterialien und Daten für die Forschung zur Verfügung zu stellen. Ihre Teilnahme ist freiwillig. Soweit Sie sich nicht beteiligen möchten oder Ihre Zustimmung später widerrufen möchten, erwachsen Ihnen daraus keine Nachteile.**

Im Folgenden informieren wir Sie über die Ziele der HUB, welche Mitglied der zentralen Biobank des DZLs ist, sowie über die Verfahrensweisen und die Maßnahmen zum Schutz Ihrer personenbezogenen Daten, damit Sie sich auf dieser Grundlage Ihre eigene Meinung bilden und eine Entscheidung treffen können.

1. Ziele der Biobank

Die HUB der MHH dient als Mitglied der DZL-Biobank der Förderung medizinischer Forschung. In der Biobank werden Biomaterialien und ausgewählte medizinische Daten langfristig aufbewahrt und für die Erforschung von Lungenerkrankungen zur Verfügung gestellt, um die Vorbeugung, Erkennung und Behandlung von Lungenerkrankungen zu verbessern. **Das Ziel dieser Forschung ist nicht, bei Ihnen oder anderen einzelnen Personen eine Diagnose zu erstellen oder krankheitsauslösende Veranlagungen nachzuweisen.**

2. Um welche Art von Biomaterialien und Daten handelt es sich?

In der Biobank werden Biomaterialien wie Blut, Lungengewebe (Biopsien sowie chirurgisch entnommenes Lungengewebe), Sputum, Spülflüssigkeiten der Atemwege (sog. Bronchoalveoläre Lavagen), exhalative Atemkondensate, Urin, Zellen, Abstriche und Luftproben gesammelt.

Bei dem Biomaterial handelt es sich um Gewebe und Körperflüssigkeiten, die im Laufe Ihres früheren derzeitigen oder zukünftigen Krankenhausaufenthaltes

a) zum Zweck der Untersuchung / Behandlung entnommen, dafür jedoch nicht mehr benötigt werden und daher ansonsten vernichtet würden. (Reste von Blutproben, Spülflüssigkeiten, Gewebeproben etc.).

b) zusätzlich bei der Routinediagnostik entnommen werden. Hierzu werden im Rahmen der Routine-Blutentnahme bis zu 25 ml Blut (etwa 5 Teelöffel) zusätzlich entnommen.

A L I D A . A U F K

* Im Rahmen dieses Textes schließt die männliche Bezeichnung stets die weibliche Bezeichnung mit ein.

Erhebung personenbezogener Daten:

Die erhobenen Daten umfassen ausgewählte Informationen zu Ihrer Person. Dabei werden sowohl medizinische Daten (z.B. Alter, Geschlecht, verordnete Medikamente, familiäre Vorbelastung für bestimmte Erkrankungen, bekannte genetische Veränderungen) als auch Untersuchungsbefunde ihres früheren, derzeitigen oder zukünftigen Krankenhausaufenthaltes erfasst (z.B. Röntgenbilder, Ultraschallbefunde, Laborwerte, Lungenfunktionen).

Die HUB und das DZL strebt grundsätzlich an, an mehreren Zeitpunkten die oben erwähnten Biomaterialien und begleitende medizinischen Daten zu erheben. Wir werden daher auch bei späterer Vorstellung in unserem Haus die o.g. Proben und erhobenen medizinischen Daten sammeln.

3. Wie werden die Biomaterialien und Daten verwendet?

Wir fragen Sie nach einer sehr breit gefassten Erlaubnis zur Verwendung Ihrer Biomaterialien und Daten. Diese werden ausschließlich für die medizinische Forschung bereitgestellt, die die Vorbeugung, Erkennung und Behandlung von Erkrankungen verbessern soll. Sie sollen im Sinne eines möglichst großen Nutzens für die Allgemeinheit für viele verschiedene medizinische Forschungszwecke verwendet werden. Informationen über die Aktivitäten des DZL auf dem Gebiet der Lungenforschung und die medizinischen Forschungsprojekte mit Biomaterialien aus den DZL-Biobanken finden Sie auf der DZL-Homepage unter www.dzl.de. Sollten Sie hierunter Forschungsvorhaben finden, deren Inhalte nicht Ihre Zustimmung finden, können Sie jederzeit ihre Einwilligung widerrufen.

Aus einem kleinen Stück Ihres Restgewebes (z.B. nach Operation bzw. endoskopischer Biopsie und histologischer Begutachtung) oder aus Ihrem Blut, sollen nach Eignungsprüfung gegebenenfalls auch Gewebe und/oder Zellkulturen angezüchtet werden. Nach erfolgreicher Kultivierung sollen verschiedene Zellfunktionen wie Wachstum und Widerstandsfähigkeit sowie die Bildung wichtiger Proteine untersucht werden, um deren Bedeutung für die Steuerung verschiedener Zellfunktionen besser zu verstehen.

Die angezüchteten Zellen sollen für spätere Versuche gelagert werden, um auch zukünftige Fragestellungen beantworten zu können. Ziel aller Untersuchungen ist es, ein besseres Verständnis über molekularbiologische Vorgänge bei Entstehung, Wachstum und Ausbreitung von Lungenerkrankungen zu erreichen und ggf. neue Therapieansätze zu finden.

Zum derzeitigen Zeitpunkt können noch nicht alle zukünftigen medizinischen Forschungsziele beschrieben werden. Diese können sich sowohl auf Lungenerkrankungen als auch auf heute zum Teil noch unbekannte Krankheiten und genetische Defekte, auch anderer Organsysteme, beziehen. Weil sich in der Forschung immer wieder neue Fragen ergeben, kann es also sein, dass Ihre Proben und Daten auch für medizinische Forschungsfragen verwendet werden, die man heute noch nicht absehen kann.

Deshalb werden an Ihren Biomaterialien möglicherweise auch genetische Untersuchungen, also Untersuchungen der Erbsubstanz, durchgeführt, und zwar unter Umständen auch eine Untersuchung Ihres gesamten Genoms. Aus Genomdaten kann möglicherweise ein Bezug zu Ihrer Person abgeleitet werden. Wir stellen jedoch technisch und organisatorisch sicher, dass Ihre Genomdaten nicht missbräuchlich verwendet werden.

Die Biomaterialien und Daten sollen langfristig aufbewahrt und für die medizinische Forschung, wie auf der Homepage des DZL (www.dzl.de) dargestellt, bereitgestellt werden.

Die Dauer der Lagerung von Biomaterialien/Speicherung von Daten richtet sich dabei an die wissenschaftliche Erforderlichkeit und die tatsächliche Verwendbarkeit der Probe. Nach dem jetzigen Stand der Wissenschaft und der Technik erscheint eine Lagerung von flüssigen Biomaterialien wie Serum, Plasma oder Lungenspülflüssigkeit nur für einen Zeitraum von etwa 80 Jahren sinnvoll. DNA-haltige Biomaterialien wie Zellen, Gewebestücke oder Paraffinschnitte können allerdings auch noch nach Jahrtausenden zuverlässig analysiert werden und wertvolle Beiträge zu wissenschaftlichen Fragestellungen leisten. Solange also die entsprechenden kollaborativen Wissenschaftsstrukturen wie das DZL (oder Folgeeinrichtungen) bestehen und aktiv an den gesammelten Daten oder Proben wissenschaftlich arbeiten, werden zumindest DNA-haltige Proben samt der dazu gehörigen klinischen Daten langfristig aufbewahrt werden. Ähnliches gilt auch für flüssige Biomaterialien, wenn sich der Stand der Technik weiterentwickelt und mit neuen Analyseverfahren auch über 80 Jahre alte Proben zuverlässig untersucht werden können.

Aus logistischen Gründen ist es der Biobank nicht möglich, individuelle Eingrenzungen (z.B. Ausschluss bestimmter Forschung, Ausschluss der Weitergabe der Materialien an Dritte) vorzunehmen. Wenn Sie mit

der oben beschriebenen Art und Dauer der Nutzung nicht in vollem Umfang einverstanden sind, werden Ihre Biomaterialien und Daten nicht in die HUB aufgenommen.

Mit der Koordinierung bzw. dem Management der HUB ist aktuell beauftragt:

**Name**	Prof. Dr. Thomas Illig
**Stelle / Institution**	Hannover Unified Biobank (HUB), MHH
**Straße / Nr.**	Feodor-Lynen-Str.15
**PLZ / Ort**	30625 Hannover

4. Welche Risiken sind mit Ihrer Spende verbunden?

a. Gesundheitliche Risiken:

Da wir für die Biobank von Patienten lediglich Körpermaterial verwenden wollen, das im Rahmen der bei Ihnen vorgesehenen diagnostischen oder therapeutischen Maßnahmen ohnehin entnommen wird und als Restmaterial normalerweise vernichtet würde, ist die Spende für Sie mit keinem zusätzlichen gesundheitlichen Risiko verbunden.

Falls im Rahmen einer routine-diagnostischen Maßnahme z.B. einer Blutentnahme *zusätzlich* bis zu 25ml Blut (das entspricht etwa 5 Teelöffeln) abgenommen wird, ist diese Entnahme für Sie mit keinem zusätzlichen gesundheitlichen Risiko verbunden.

b. Weitere Risiken:

Bei jeder Erhebung, Speicherung und Übermittlung von Daten aus Ihren Biomaterialien im Rahmen von Forschungsprojekten bestehen Vertraulichkeitsrisiken (z.B. die Möglichkeit, Sie zu identifizieren), insbesondere im Hinblick auf die Information zu Ihrer Erbsubstanz. Diese Risiken lassen sich nicht völlig ausschließen und steigen, je mehr Daten miteinander verknüpft werden können, insbesondere auch dann, wenn Sie selbst (z.B. zur Ahnenforschung) genetische Daten im Internet veröffentlichen.

Die HUB versichert Ihnen, alles nach dem Stand der Technik Mögliche zum Schutz Ihrer Privatsphäre zu tun. Näheres finden Sie unter Punkt 7: „Wer hat Zugang zu Ihren Biomaterialien und Daten?“.

5. Welcher Nutzen ergibt sich für Sie persönlich?

Persönlich können Sie für Ihre Gesundheit keinen unmittelbaren Vorteil oder Nutzen aus der Spende Ihrer Proben und Daten erwarten. Deren Auswertung dient ausschließlich Forschungszwecken und nicht dazu, Rückschlüsse auf Ihre Gesundheit zu ziehen. Eine Rückmeldung individueller Forschungsergebnisse an Sie erfolgt nicht.

6. Welcher Nutzen ergibt sich für unsere Gesellschaft?

Alle derzeit durchgeführten wie auch künftige medizinisch-wissenschaftliche Forschungsvorhaben zielen auf eine Verbesserung unseres Verständnisses für die Krankheitsentstehung und die Diagnosestellung und auf dieser Basis auf die Neuentwicklung von verbesserten Behandlungs- und Vorbeugungsmaßnahmen. Informationen über die Aktivitäten des DZL und der DZL-Biobanken auf dem Gebiet der Lungenforschung finden Sie auf der DZL-Homepage unter www.dzl.de.

7. Wer hat Zugang zu Ihren Biomaterialien und Daten und wie werden sie geschützt?

a. Kodierung Ihrer Biomaterialien und Daten

Alle Daten, die Ihre Person unmittelbar identifizieren (Name, Geburtsdatum, Anschrift etc.) werden unverzüglich nach Gewinnung der Biomaterialien durch die HUB durch einen Code ersetzt (pseudonymisiert, verschlüsselt). Bei der Überführung der Datensätze und Proben an die DZL-Biobank wird der Datensatz nochmals neu kodiert und gespeichert. Diese doppelte Kodierung schließt eine Identifizierung Ihrer Person durch Unbefugte nach heutiger Kenntnis weitestgehend aus. Erst in dieser Form werden die Biomaterialien und Daten für Forschungszwecke innerhalb des DZL zur Verfügung gestellt.

Die Sie unmittelbar identifizierenden Daten bleiben in der Einrichtung, in der die Proben und Daten gewonnen wurden, und werden dort getrennt von den Biomaterialien und medizinischen Daten gespeichert. Dabei werden technische und organisatorische Maßnahmen (z.B. geschützter Serverbereich, Firewall, Datenhosting an getrennten universitären Rechenzentren, Kodierung der Daten nach dem Prinzip der Public-Key-Kryptographie) getroffen, um einen missbräuchlichen Zugriff auf die Daten zu verhindern.

Ein Zugriff auf sie erfolgt nur, um entweder weitere oder fehlende medizinische Daten aus Ihren Krankenunterlagen zu ergänzen oder erneut mit Ihnen in Kontakt zu treten, falls Sie der Kontaktaufnahme zugestimmt haben (s. unten, Punkt 9). Eine Weitergabe der personenbezogenen Daten an Forscher oder andere unberechtigte Dritte, etwa Versicherungsunternehmen oder Arbeitgeber, erfolgt nicht.

b. Weitergabe von Biomaterialien und Daten

Die doppelt verschlüsselten Biomaterialien und medizinischen Daten können für genauer bestimmte medizinische Forschungszwecke auf Antrag und nach zuvor festgelegten Regeln an andere Einrichtungen wie Universitäten, Forschungsinstitute und forschende Unternehmen innerhalb der EU weitergegeben werden.

Dabei werden die Daten unter Umständen auch mit medizinischen Datensätzen in anderen

Datenbanken verknüpft, sofern die gesetzlichen Voraussetzungen hierfür erfüllt sind. Biomaterialien und Daten, die an Forscher herausgegeben wurden, dürfen nur für den beantragten Forschungszweck verwendet und vom Empfänger nicht zu anderen Zwecken weitergegeben werden. Nicht verbrauchtes Material wird an die Biobank zurückgegeben oder vernichtet.

c. Die Weitergabe in Länder außerhalb der Europäischen Union

Ihre Proben und Daten können auch an Forschungspartner in Ländern außerhalb der EU. weitergegeben werden, wenn eine der folgenden Voraussetzungen erfüllt ist:

- Die Europäische Kommission hat bei dem Land ein angemessenes gesetzliches Datenschutzniveau festgestellt,
  oder, wenn dies nicht erfolgt ist,
- Die MHH vereinbart mit den Forschungspartnern vertragliche Datenschutzklauseln, die von der Europäischen Kommission oder der zuständigen Aufsichtsbehörde beschlossen oder genehmigt wurden. Sie können bei der MHH eine Kopie dieser Datenschutzklauseln erhalten.

Darüber hinaus kann es aber auch vorkommen, dass Proben und Daten an Forschungspartner in Drittländern weitergegeben werden sollen, für die keine dieser beiden Voraussetzungen erfüllt ist. Diese Länder haben **möglicherweise ein niedrigeres Datenschutzniveau als die EU.** Die HUB sichert zu, auch in diesen Fällen die Forschungspartner vertraglich, soweit rechtlich möglich, zur Einhaltung des EU-Datenschutz-Niveaus zu verpflichten. Dennoch besteht das Risiko, dass staatliche oder private Stellen auf Ihre Daten zugreifen, obwohl dies nach dem europäischen Datenschutzrecht nicht zulässig wäre. Zudem kann es sein, dass Ihnen dort weniger oder schlechter durchsetzbare Betroffenenrechte zustehen und es keine unabhängige Aufsichtsbehörde gibt, die Sie bei der Wahrnehmung ihrer Rechte unterstützen könnte. **Eine Weitergabe Ihrer Proben und Daten kann in diesem Fall nur erfolgen, wenn Sie dem ausdrücklich zugestimmt haben. Dazu können Sie in der Einwilligungserklärung das entsprechende Kästchen ankreuzen.**

d. Bewertung durch eine Ethik-Kommission

Voraussetzung für die Verwendung der Biomaterialien und Daten für ein konkretes medizinisches Forschungsprojekt innerhalb oder außerhalb des DZL ist, dass das Forschungsvorhaben inhaltlich durch entsprechende unabhängige Gremien (z.B. eine Ethikkommission) bewertet wurde. Die Ethikkommission prüft die ethischen und rechtlichen Aspekte des Forschungsprojekts. Nur wenn diese keine Einwände oder ethisch-rechtliche Bedenken dagegen hat, wird das konkrete Forschungsvorhaben auch durchgeführt.

e. Veröffentlichungen

Wissenschaftliche Veröffentlichungen von Ergebnissen erfolgen ausschließlich anonymisiert, also in einer Form, die keine Rückschlüsse auf Ihre Person zulässt. Das gilt insbesondere auch für genetische Informationen. Möglich ist allerdings eine Aufnahme genetischer Informationen in besonders geschützte wissenschaftliche Datenbanken, die für die Allgemeinheit nicht zugänglich sind.

8. Erlangen Sie oder die Biobank einen finanziellen Vorteil aus der Nutzung Ihrer Biomaterialien und Daten?

Mit der Überlassung der Biomaterialien an die MHH, die lokal die DZL-Biobank vertritt, werden diese Eigentum der MHH. Ferner ermächtigen Sie die MHH, Ihre Daten zu nutzen.

Für die Überlassung Ihrer Biomaterialien und Daten erhalten Sie kein Entgelt. Sollte aus der Forschung ein kommerzieller Nutzen erzielt werden, werden Sie daran nicht beteiligt. Die Biobank verwendet Ihre Biomaterialien und Daten ausschließlich für wissenschaftliche Zwecke Die Proben und Daten werden nicht

verkauft. Für die Bereitstellung der Proben und Daten kann die Biobank jedoch von den Nutzern eine Aufwandsentschädigung erheben.

9. Erfolgt eine erneute Kontaktaufnahme mit Ihnen?

Zur Erhebung von weiteren Verlaufsdaten kann es sinnvoll werden, zu einem späteren Zeitpunkt erneut Kontakt mit Ihnen aufzunehmen, um ergänzende Informationen und/oder Biomaterialien von Ihnen zu erbitten. Zudem kann die erneute Kontaktaufnahme genutzt werden, um z. B. Ihre Einwilligung zum Abgleich mit anderen Datenbanken einzuholen. Sollte eine solche Kontaktaufnahme von Ihnen erwünscht sein, wird dies durch unsere Klinik erfolgen.

Kreuzen Sie in der Einwilligungserklärung bitte an, ob Sie eine erneute Kontaktaufnahme in diesen Fällen wünschen oder nicht.

10. Was beinhaltet Ihr Widerrufsrecht?

Sie können Ihre Einwilligung zur Verwendung Ihrer Biomaterialien und Daten jederzeit ohne Angabe von Gründen und ohne nachteilige Folgen für Sie widerrufen. Die Rechtmäßigkeit der bis zum Widerruf erfolgten Nutzung der Proben und Daten bleibt davon jedoch unberührt. Im Falle eines Widerrufs können Sie entscheiden, ob Ihre Biomaterialien vernichtet und die dazu gehörenden Daten gelöscht werden sollen. Eine Datenlöschung kann allerdings nur erfolgen, soweit dies mit zumutbarem technischem Aufwand möglich ist. Zudem können Daten aus bereits durchgeführten Analysen nicht mehr entfernt werden. Statt der Vernichtung bzw. Löschung können Sie auch zustimmen, dass die Biomaterialien und Daten in anonymisierter Form für weitere Forschungsvorhaben verwendet werden dürfen. Anonymisierung bedeutet, dass der Identifizierungscode gelöscht wird, über den ermittelt werden kann, von welcher Person die Probe stammt. Sobald der Bezug der Biomaterialien und der übrigen Daten zu Ihrer Person gelöscht wurde, ist eine gezielte Vernichtung aufgrund Ihrer Entscheidung jedoch nicht mehr möglich. Zudem können bereits für Forschungsprojekte ausgegebene Proben nicht zurückgeholt und Daten aus bereits durchgeführten Analysen nicht mehr entfernt werden. Trotz Widerrufs kann eine spätere Zuordnung des genetischen Materials zu Ihrer Person über andere Quellen niemals ausgeschlossen werden.

Wenden Sie sich für einen Widerruf bitte an die unter Punkt 3 genannte Leitung der Biobank mit den dort angegebenen Kontaktdaten. Sie wird ihren Widerruf an die zuständige Datentreuhänderstelle weiterleiten.

11. Welche weiteren Datenschutzrechte haben Sie?

Rechtsgrundlage für die Datenverarbeitung ist Ihre Einwilligung gemäß Art. 6 Abs. 1 Buchst. a und Art. 9 Abs. 2 Buchst. a der EU-Datenschutz-Grundverordnung (EU-DSGVO). Nach Art. 13ff EU-DSGVO haben Sie das Recht vom Verantwortlichen für die Erhebung personenbezogener Daten Auskunft über die von Ihnen gespeicherten personenbezogenen Daten zu verlangen. Ebenso können Sie eine Berichtigung falscher Daten, eine Übertragung der von Ihnen zur Verfügung gestellten Daten sowie eine Löschung der Daten oder Einschränkung ihrer Verarbeitung verlangen. Ihre Daten werden im Falle der HUB durch die MHH im Falle der DZL Biobank durch das Zentrum für Lungenforschung e.V. (www.dzl.de; Koordinator aktuell Prof. Dr. W. Seeger, Sitz Gießen), verarbeitet.

Stelle / Institution Klinik für Pneumologie der MHH & DZL/BREATH
Straße / Nr. Carl-Neuberg-Straße 1
PLZ / Ort 30625 Hannover
Telefon 0511-532-3531

Verantwortliche Person für die Datenverarbeitung:
Stelle / Institution Klinik für Pneumologie der MHH & DZL/BREATH
Prof. Dr. med. Tobias Welte, Klinik für Pneumologie,
Prof. Dr. Thomas Illig, Hannover Unified Biobank (HUB)
Straße / Nr. Carl-Neuberg-Straße 1
PLZ / Ort 30625 Hannover
Telefon 0511-532-3531 / Biobank: 0511-5350-8451

Die MHH hat einen Datenschutzbeauftragten bestellt. An ihn können Sie sich mit allen Anliegen zur Datenverarbeitung und zur Einhaltung des Datenschutzes wenden. Seine Kontaktdaten lauten wie folgt:

Datenschutzbeauftragte/r:
Stelle / Institution Datenschutzbeauftragte/r der MHH
Straße / Nr. Carl-Neuberg-Straße 1
PLZ / Ort 30625 Hannover
Telefon 0511-532-2555

Zudem haben Sie ein Beschwerderecht bei der Datenschutzaufsichtsbehörde, wenn Sie meinen, dass die Verarbeitung Ihrer Daten datenschutzrechtlich nicht zulässig ist (Art. 77 DSGVO). In Ihrem Falle handelt es sich dabei um folgende Institution:

Zuständige Datenschutzaufsichtsbehörde:
Stelle / Institution Landesbeauftragte/r für den Datenschutz Niedersachsen
Straße / Nr. Prinzenstraße 5
PLZ / Ort 30159 Hannover
Telefon 0511-120-4500

12. Wo kann ich weitere Informationen erhalten?

Sollte Ihnen etwas unklar sein, fragen Sie bitte Ihren behandelnden Arzt bzw. Ihren Studienarzt, bevor Sie Ihre Zustimmung erteilen. Sie können sich wegen Rückfragen auch zu einem späteren Zeitpunkt an

Prof. Dr. med. Tobias Welte
Klinik für Pneumologie
Carl-Neuberg-Straß 1
30625 Hannover

wenden.

Bitte lesen Sie die folgende Einwilligungserklärung aufmerksam durch, kreuzen Sie Zutreffendes an und unterschreiben Sie anschließend am Ende dieser Einwilligungserklärung, sofern Sie einverstanden sind.

EINWILLIGUNGSERKLÄRUNG

Name:	Vorname:	Geburtsdatum:

Ich bin damit einverstanden, dass meine Biomaterialien und Daten, wie in der Patienteninformation beschrieben, an die Hannover Unified Biobank (HUB) der Medizinischen Hochschule Hannover, der lokalen DZL-Biobank, gegeben und für medizinische Forschungszwecke aufbewahrt und verwendet werden.

Dies betrifft unter Umständen auch die (doppelt kodierte) Weitergabe für Forschungsprojekte oder an Forschungseinrichtungen im Ausland mit möglicherweise niedrigerem Datenschutzniveau. Es werden nur medizinische, jedoch keine meine Person identifizierende Daten weitergegeben.

Ich stimme der Entnahme von Blut-, Gewebe bzw. der unter Punkt 2 angegebenen Biomaterialien zu. Ich stimme zu, dass aus meinen Biomaterialproben gegebenenfalls Zellkulturen kultiviert werden. Ich stimme zu, dass meine Biomaterialproben und die darin enthaltenen Bestandteile wie DNA, RNA und Proteine, über molekularbiologische Methoden analysiert werden, einschließlich einer kompletten Analyse des Erbgutes.

Das Eigentum an den Biomaterialien übertrage ich an die lokal mit der Biomaterial Sammlung beauftragten HUB. Ich weiß, dass ich über die Teilnahme an der Biobank keinen kommerziellen Nutzen oder persönliche Vorteile ziehen werde. Ich verzichte zugleich auf Entgeltzahlungen und urheber- oder patentrechtliche Ansprüche, die z.B. die mit der Entwicklung neuer Therapien oder Diagnostika verbunden sind.

Ich habe die Patienteninformation gelesen und hatte die Gelegenheit, Fragen zu stellen.

Ich weiß, dass meine Teilnahme freiwillig ist und ich meine Einwilligung jederzeit ohne Angabe von Gründen widerrufen kann, ohne dass mir daraus irgendwelche Nachteile entstehen. Bei Widerruf werden meine personenbezogenen Daten anonymisiert. Die Datenverarbeitung bis zum Zeitpunkt meines Widerrufs bleibt rechtmäßig.

Ich bin damit einverstanden, dass ich bzw. mein Hausarzt evtl. zu einem späteren Zeitpunkt erneut kontaktiert wird (falls nicht gewünscht, bitte „nein“ ankreuzen).

- zum Zweck der Gewinnung weiterer Informationen / Biomaterialien, ☐ **nein**

- zum Zweck der Einwilligung in den Abgleich mit anderen Datenbanken, ☐ **nein**

Diese Rückmeldung soll erfolgen über die Einrichtung, in der meine Biomaterialien / Daten gewonnen wurden oder über folgenden Arzt (falls gewünscht, bitte angeben)

Name und Anschrift des Arztes: ....

Einwilligung zur Erhebung, Verarbeitung und Nutzung personenbezogener Daten

Ich erkläre mich damit einverstanden, dass die Hannover Unified Biobank (HUB) und die DZL Biobank wie in der Patienteninformation beschrieben,

- personenbezogene Daten von mir erhebt und speichert,
- weitere Angaben über meine Gesundheit aus meinen Krankenunterlagen (wie z.B. Lungenfunktions- und Laborwerte, Ultraschallbefunde, Röntgenbilder) aus meinen Krankenunterlagen entnimmt,
- und die Daten gemeinsam mit meinen Biomaterialien pseudonymisiert (das heißt kodiert) speichert bzw. aufbewahrt.

Die Biomaterialien und Daten dürfen langfristig für medizinische Forschungsvorhaben verwendet werden. Eine Übersicht über die Forschungsvorhaben unter Verwendung von Biomaterialien finde ich auf der Homepage des DZL unter www.dzl.de.

Sie dürfen pseudonymisiert nach Prüfung und Beratung durch die zuständige öffentlich- rechtliche Ethikkommission an Universitäten, Forschungsinstitute und forschende Unternehmen, ggf. auch im Ausland, zu Zwecken medizinischer Forschung weitergegeben werden. Meine Person identifizierende Daten werden nicht weitergegeben.

Meine Einwilligung umfasst auch die Übermittlung meiner pseudonymisierten, d.h. verschlüsselten Patientendaten für medizinische Forschungszwecke in Länder, bei denen von der Europäischen Kommission kein angemessenes Datenschutzniveau festgestellt wurde. Dies ist generell zulässig, wenn ein Angemessenheitsbe schluss der Europäischen Kommission vorliegt oder behördlich genehmigte Datenschutzklauseln angewendet werden.

Darüber hinaus willige ich in die Weitergabe meiner Biomaterialien und Daten in Länder außerhalb der EU auch in den Fällen ein, in denen kein Angemessenheitsbeschluss der Europäischen Kommission vorliegt und keine behördlich genehmigten Datenschutzklauseln angewendet werden. Über die möglichen Risiken einer solchen Weitergabe bin ich aufgeklärt worden.

(Ziff. 7c in der Information). ☐ Ja ☐ Nein

Ich bin darüber aufgeklärt worden, dass unter Umständen auch Analysen des kompletten Erbgutes (Genom) durchgeführt werden und aus Genomdaten grundsätzlich ein Bezug zu meiner Person hergestellt werden könnte, dass das DZL aber technische und organisatorische Maßnahmen gegen eine missbräuchliche Verwendung getroffen hat.

Ich bin darüber aufgeklärt worden, dass ich meine Einwilligung gegenüber der HUB bzw. der DZL Biobank ohne Angabe von Gründen jederzeit widerrufen kann. Beim Widerruf werden auf mein Verlangen die verbliebenen Biomaterialien vernichtet und die erhobenen Daten gelöscht oder anonymisiert. Daten aus bereits durchgeführten Analysen können nicht mehr entfernt oder gelöscht werden.

Ich habe das Recht, Auskunft (einschließlich unentgeltlicher Überlassung einer Kopie) über die mich betreffenden personenbezogenen Daten zu erhalten sowie ggf. deren Berichtigung zu verlangen. Bei Vorliegen der gesetzlichen Voraussetzungen kann ich die Löschung personenbezogener Daten und die Einschränkung der Verarbeitung verlangen, sowie Widerspruch gegen die Verarbeitung einlegen. Wenn ich diese Rechte gegenüber der Datenverarbeitenden Stelle geltend mache, wird geprüft, ob die gesetzlichen Voraussetzungen erfüllt sind. Eine Kopie der Patienteninformation und Einwilligungserklärung habe ich erhalten. Das Original verbleibt in der MHH.

_________________________________ _________________________________

Name des Patienten *(in Druckbuchstaben)* Ort, Datum Unterschrift des Patienten

Ich habe das Aufklärungsgespräch geführt und die Einwilligung des Patienten eingeholt.

_________________________________ _________________________________

Name des Arztes *(in Druckbuchstaben)* Ort, Datum Unterschrift des Arztes
